# Novel Diagnostic Methods for Infective Endocarditis

**DOI:** 10.3390/ijms25021245

**Published:** 2024-01-19

**Authors:** Anna Burban, Dorota Słupik, Aleksandra Reda, Ewa Szczerba, Marcin Grabowski, Agnieszka Kołodzińska

**Affiliations:** 1 1st Chair and Department of Cardiology, Medical University of Warsaw, 02-097 Warsaw, Poland; 2 Doctoral School, Medical University of Warsaw, 02-091 Warsaw, Poland

**Keywords:** infective endocarditis, PET/CT tracers, 16S/18S rRNA PCR, molecular diagnosis

## Abstract

Infective endocarditis (IE) remains a dangerous disease and continues to have a high mortality rate. Unfortunately, despite continuous improvements in diagnostic methods, in many cases, blood cultures remain negative, and the pathogen causing endocarditis is unknown. This makes targeted therapy and the selection of appropriate antibiotics impossible. Therefore, we present what methods can be used to identify the pathogen in infective endocarditis. These are mainly molecular methods, including PCR and MGS, as well as imaging methods using radiotracers, which offer more possibilities for diagnosing IE. However, they are still not widely used in the diagnosis of IE. The article summarizes in which cases we should choose them and what we are most hopeful about in further research into the diagnosis of IE. In addition, registered clinical trials that are currently underway for the diagnosis of IE are also presented.

## 1. Introduction

Infective endocarditis (IE) is a disease of the endocardial surface of the heart. The infection is usually associated with heart valves (native or prosthetic) or implanted cardiac devices. The incidence of IE is currently about 13.8 cases per 100,000 per year [1]. Approximately 25–30% of newly reported IE cases are healthcare-associated infections. IE is associated with a mortality rate of up to 30% within 30 days [2].

Risk factors for IE include previous infective endocarditis; valvular heart disease; the presence of prosthetic heart valves, central venous, or arterial catheter; a transvenous implantable electronic device; congenital heart defects; immunosuppression; hemodialysis; recent dental or surgical procedures, or hospitalization and intravenous drug use. Rheumatic heart disease remains a predisposing risk factor in developing countries. The population at risk for IE continues to grow as the number of patients following cardiovascular interventions increases. Moreover, the number of people living with congenital heart defects has increased significantly, mainly due to improvements in the diagnosis and treatment of children with congenital heart defects and advances in cardiac surgery. However, native valve endocarditis (NVE) is still the most common, but the percentage of prosthetic valve endocarditis (PVE) is increasing [3].

IE continues to be a diagnostic challenge, and the main reason for this is the highly variable clinical presentation of patients with IE. It should be considered in all patients with sepsis or fevers of unknown origin in the presence of risk factors. Unfortunately, there is still a significant percentage of patients in the “possible IE” category. In addition, the presence of prosthetic valves reduces the sensitivity of these criteria and requires the use of newer imaging studies.

Transthoracic echocardiography (TTE) is the mainstay of imaging methods in the diagnosis of IE and should be performed in all cases of suspected endocarditis (Class I of ESC Guidelines 2023) [1]. Imaging cardiac signs may include valve vegetation, abscess, the expansion of perivalvular infection, and heart failure due to valve dysfunction [4]. Transesophageal echocardiography (TEE) plays an important role in diagnosing IE (when TTE is negative), characterizing lesions, and identifying local complications. TEE also has a much higher sensitivity in patients with valve prostheses. Alternative imaging modalities are playing an increasingly important role in the diagnosis and treatment of IE. Computed tomography (CT), nuclear imaging, and magnetic resonance imaging (MRI) are now part of the diagnostic strategy for suspected IE, as they can help evaluate local IE complications as well as IE-related distant lesions. Moreover, they can identify the primary source of bacteremia in patients who develop secondary IE [5]. PET scanning in IE is based on the uptake of 18-fluorodeoxyglucose (FDG) by cells, such as leukocytes and monocytes, at the site of infection. In addition, [18-F]FDG-PET/CT imaging can be helpful in evaluating cardiac device infection.

**Figure 1:**
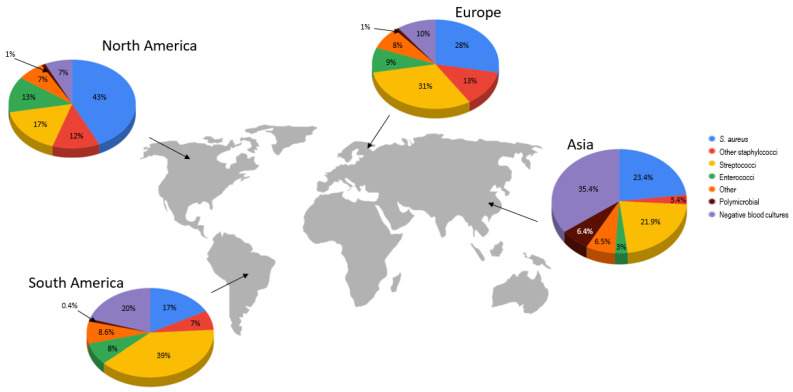
Etiology of infective endocarditis in North America, South America, Europe, and Asia [6,7].

IE is caused by various micro-organisms, mainly bacteria (>90% of IE), as well as fungi (<1%), and, very rarely, rickettsiae, chlamydiae, and mycoplasmas. Rarely, non-IE, including nonbacterial thrombotic (marantic) endocarditis, can be caused by autoimmune or neoplastic conditions. The etiology of IE is not homogeneous worldwide due to the peculiarities of a particular geographic region and different risk factors for infection. Figure 1 shows the prevalence of individual bacteria causing infective endocarditis in each continent: Europe, North America, South America, and from regions classified as other (Australia, Asia, and Africa countries) [6]. *Staphylococcus aureus* is the most common pathogen in North America and Europe, while in South America, it is *Streptococcus viridans*, followed by culture-negative findings and *Staphylococcus aureus* [6]. The etiology of IE, according to these data, is presented in Figure 1. However, there are no representative data from the Asian population. In the study describing the IE in the Chinese population, the most frequent pathogen was *Stapylococcus aureus* (23.4%), followed by streptococci (21.9%) [7].

However, the etiology of IE has been changing over the years. *Streptococcus viridans* used to be the most common cause of IE associated with bacteremia after dental procedures, but this has changed due to recommendations for anti-microbial prophylaxis. Importantly, limiting antibiotic prophylaxis to only high-risk patients does not result in an increased incidence of streptococcal IE [8]. Currently, *Staphylococcus aureus* is the leading cause of IE in most countries around the world, occurring in about 31% of cases [9]. Staphylococcal species, including methicillin-resistant strains (MRSA), have been observed to be increasingly associated with medical procedures and healthcare contact [8,10]. In addition, the incidence of IE with enterococcal etiology is increasing, especially in the elderly [2]. A retrospective study involving 158 patients diagnosed with IE was conducted in Japan. It aimed to compare healthcare-associated IE (HAIE) and community-acquired IE (CAIE) [9]. HAIE was classified for 33.5% of IE cases [9]. *Staphylococcus aureus* was found to be more prevalent in patients with HAIE than CIE (32.1% vs. 14.3%; *p* = 0.01), and methicillin-resistant staphylococci were detected in 15.1% of patients with HAIE compared to 1.0% of patients with CIE (*p* = 0.0004). Conversely, the percentage of streptococci was significantly higher in patients with CIE than HAIE (62.9% vs. 24.5%; *p* < 0.0001) [9]. In addition, patients with HAIE had a higher in-hospital mortality rate (32.1% vs. 4.8%; *p* < 0.0001). In fungal IE, about half of the cases are caused by Candida fungi. According to the EURO-ENDO registry, none of the IE etiologies was an independent factor in mortality [2].

Optimal therapy consists of anti-microbial treatment with the long-term (>4 weeks) use of antibacterial agents to eliminate the infection. However, for the proper use of anti-microbial therapy, truthful and accurate laboratory diagnosis is essential. Therefore, summarizing diagnostics in IE will be the subject of this review, including laboratory diagnosis using molecular methods, as well as novel potential diagnostic methods with the use of PET/CT scanning and novel PET tracers.

## 2. Diagnosis of IE

The diagnosis is based on clinical suspicion, which is supported by microbiological data and imaging, as included in the latest ESC guidelines [1]. Suspicion of IE is usually due to fever, new heart murmurs, and positive blood cultures if an alternative focus of infection is not known. This is especially true in patients with one or more risk factors. In the latest ESC guidelines, it is recommended that the endocarditis team be involved as soon as possible to help further manage patients with suspected IE [1].

### 2.1. Clinical Features

Infective endocarditis has a highly variable clinical course. It can present as an acute, rapidly progressive infection but also as a chronic disease, even without fever and nonspecific symptoms. Infective endocarditis can also present with complications that mimic many other conditions (such as rheumatologic, neurologic, and autoimmune diseases, and even malignancies). According to the registry, fever (77.7%), cardiac murmur (64.5%), and congestive HF (27.2%) were the most frequent clinical presentations [11]. Atypical presentation is common in elderly or immunocompromised patients [12,13,14]. In order to avoid delays in diagnosis in these and other high-risk groups, such as people with congenital heart defects (CHDs) or valve prostheses, IE should be ruled out as soon as possible in these groups [15]. In children and adolescents, the incidence of IE is much lower, with only 0.05–0.12/1000 cases in hospitalized pediatric patients, mostly as a complication of a pre-existing CHD [10]. The overall mortality rate due to IE among children and adolescents is 16–25% [10]. According to the population-based analysis, the cumulative incidence of IE in children with CHD is 6.1/1000 children (95% confidence interval, 5.0–7.5) [16]. Viridans-type streptococci and *Staphylococcus aureus* remain the leading causative agents for endocarditis in pediatric and CHD patients [8,10,17]. As adults, CHD patients with IE are younger and have fewer comorbidities compared to patients with IE without CHDs from an ESC-EORP-EURO-ENDO study [17]. Interestingly, both short-term and long-term prognosis are better in CHD patients than in non-CHD IE patients; however, it can be partly explained by their younger age in the time of IE [17]. The incidence of IE is also higher in a population of solid organ transplant recipients compared to the general population. Staphylococci and enterococci represent the most frequently isolated pathogens (more common with HAIE [18]).

In patients infected with HIV, *Staphylococcus aureus* was the most common organism isolated from blood culture, with a high incidence of MRSA (24.1%) in this group of patients [19]. However, there were no significant differences in the clinical presentation of infective endocarditis when the course of infection was compared between HIV-infected and non-HIV-infected patients. Moreover, complications were not more common in the HIV-infected patient group [19,20].

### 2.2. Microbiological Diagnosis—Blood Culture

If IE is suspected, at least three sets of blood cultures should be obtained at 30 min intervals prior to antibiotic therapy [1]. It is necessary to deliver the diagnosis and provide live bacteria for both identification and susceptibility testing. Blood cultures should be incubated in both aerobic and anaerobic atmospheres [21]. Blood for the test is drawn from the patient’s veins from different insertion sites, with at least 1 h between the first and last collection [1]. With modern automated systems used for blood cultures, it is possible to detect HACEK bacteria, as well as those species that require specific culture media. For this purpose, an extended incubation period of up to 14 days is recommended [22]. The major criteria for the diagnosis of IE are typical micro-organisms consistent with IE (Oral streptococci, *Streptococcus gallolyticus*, the HACEK group, *S. aureus*, and *Enterococcus faecalis*) from two separate blood cultures, micro-organisms consistent with IE from continuously positive blood cultures, or a single positive blood culture for *C. burnetiid* or a phase I IgG antibody titer of >1:800 [1].

**Figure 2:**
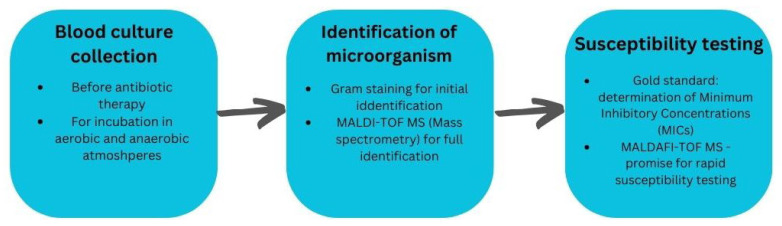
A summary of the procedure associated with the collection and examination of blood cultures.

Unfortunately, it still takes a long time between the collection of a blood sample for culture and the final identification of the organism responsible for bacteremia and antibiotic susceptibility testing. Once a positive culture result is confirmed, the first identification is based on Gram staining. However, micro-organisms that are cultured can also be identified by more specific and modern methods. Today, full identification can be achieved in a matter of hours using the matrix-assisted laser desorption/ionization MALDI-TOF MS (mass spectrometry) method. This system bases the identification of micro-organisms on mass spectrometry peptide spectra [23]. By identifying a species-specific protein, MALDI-TOF MS identifies the cultured micro-organism. However, despite technical developments and advances toward rapid susceptibility determination using MALDI-TOF MS, the gold standard for susceptibility determination is still the determination of minimum inhibitory concentrations (MICs). This is necessary for choosing the appropriate antibiotic therapy and, thus, must be carried out according to a validated, standardized methodology [24]. So far, the MALDI-TOF MS technology is the most promising type of assay for rapid and reliable susceptibility testing to replace the MIC classification [24]. The summary of the procedure associated with the collection and examination of blood cultures is shown in Figure 2.

## 3. Blood Culture-Negative Infective Endocarditis (BCNIE)

In the case of non-identified bacteria, while fulfilling the other clinical criteria of infective endocarditis, we can diagnose blood culture-negative endocarditis (BCNIE). It refers to IE in which no causative micro-organism can be grown using the usual blood culture methods, thus remaining a diagnostic and therapeutic challenge. In the case of BCNIE, further diagnosis using special methods (which will be further listed in this article) is needed. Due to the difficulty in diagnosing BCNIE, it is challenging to estimate its exact incidence. Based on a review of the available literature, the incidence of BCNIE is currently estimated to be approximately 10–20% of all endocarditis [6,25]. BCNIE most commonly arises as a consequence of sterilized blood cultures due to previous antibiotic administration. In retrospective studies, it can be responsible for 35–74% of the prevalence of BCNIE [25]. In addition, it is related to the peculiarities of a given geographic region, including the availability of antibiotics, pathogen resistance to the therapies used, the prevalence of particular strains, and the diagnostic methods used [26].

BCNIE can also be caused by fungi or bacteria for which isolation culturing on specialized media is required, and their growth is relatively slow. BCNIE, which was once mostly caused by HACEK pathogens (*Haemophilus*, *Aggregatibacter*, *Cardiobacterium*, *Eikenella*, and *Kingella*) and nutritionally variant streptococci (*Granulicatella* spp. and *Abiotrophia defectiva*), is now mainly caused by infection from pathogens such as Bortanella species, Coxiella burnetti, and fungi. These pathogens are usually very demanding and time-consuming to culture, requiring special incubation conditions as well as other specific media [27]. However, modern automated culture systems help to identify these organisms within 5 days of incubation [28,29].

Fungi account for approximately 1–2% of cases of IE [6]. *Candida albicans* is the most common cause, and it can be cultured with automated culture systems with other yeasts in routine blood culture within 2–5 days. However, the other fungi do not traditionally grow on routine blood cultures. Specific testing needs to be performed according to the local epidemiology and risk factors of patients, as those are usually the causative agents in non-immunocompetent patients. Currently, new diagnostic methods are being studied for all kinds of fungal infections. The PCR method even showed 92% sensitivity in the diagnosis of fungal endocarditis [30]. For fungal infections, both panfungal and specific-PCR can be performed for etiological diagnosis [31]. PCR-based techniques proved to be fast and sensitive and enabled definitive diagnosis in all cases in the conducted study, with the detection of a total of 28 fungal species [31].

New diagnostic methods are also available for infections caused by Candida species. One of these is the T2Candida system, in which amplified DNA targets are detected by T2 magnetic resonance. In multi-center studies, it has been shown that T2Candida is 89–91% sensitive and 98% specific for candidemia. It detects the five most common Candida species [32,33]. Galactomannan level has shown its utility in diagnosing disseminated Aspergillus infections [30]. For *Cryptococcus* infections, its antigen (CRAg) is an established diagnostic biomarker, which is more sensitive than culture [32]. For other, more rare fungal species (as *Blastomyces*, *Coccidioides* etc.) specific serology or PCR testing should be performed.

However, there are also some other bacteria that can cause BCNIE. Mycobacteria (with only some cases reported in the literature as a causative agent) cannot be routinely identified, and molecular testing is required to establish the diagnosis. *Tropheryma whipplei* can also be a causative organism in BCNIE and is usually diagnosed from cardiac tissue samples. The intracellular pathogens cannot be cultured in blood cultures. They are mainly diagnosed with the use of serology testing. The most common one is Coxiella burnetii, which was identified as a causative agent in 37–43% of cases of BCNIE [34,35]. Moreover, many cases can be caused by the Bartonella species. Other species (*Legionella* spp., *Chlamydia* spp., and *Mycoplasma* spp.) very rarely cause BCNIE. A diagnosis may be established by examining the explanted valves using histo-pathological or molecular techniques.

In the end, the cause of endocarditis can be non-infectious. The incidence of non-infective causes has been described as 2.5% of cases [34]. It is most commonly associated with systemic lupus erythematosus, rheumatoid arthritis, or metastatic malignancy. Laboratory findings of a hypercoagulable state (lupus anticoagulant, anti-cardiolipin antibodies, and anti-β2-glycoprotein 1 antibodies) may be present; however, they are non-specific [1].

When there is clinical suspicion of IE and any blood cultures remain negative after 48 h, different methods of testing should be performed. All of them will be described in the next paragraphs. Serological testing should be performed by taking into consideration the clinical characteristics of the patients and the local epidemiology. In addition, any tissue or prosthetic material obtained at surgery must be subjected to systematic culture, histological examination, and 16S or 18S rRNA sequencing aimed at documenting the presence of micro-organisms.

Currently, there are two registered clinical trials regarding blood culture in IE. First, a prospective, case-control trial (NCT03153384) aims to evaluate the performance of a single high-volume blood culture sampling strategy vs. the traditionally used multiple sampling strategy for the diagnosis of IE in a population of adults suspected of infective endocarditis. For each patient, one single high-volume blood culture (three aerobic and three anaerobic of 8 to 10 mL each, numbered) and two samples of 16 to 20 mL (one aerobic bottle and one anaerobic for each sample) are taken, and the results will be compared to see if the identified organism is the same. The second trial (NCT04257292) evaluates the possible utilization of next-generation sequencing (NGS) to predict the virulence factors of *S. aureus* and to compare this to the virulence factors determined using cell culture-based assays (CCBAs). Cultured blood and infected tissue valve samples will be examined using both NGS and CCBA. The goal of the study is to compare the results in virulence factors by using these two methods, to characterize the prevalence of different *S. aureus* strains, and to assess the potential of these methods in the diagnosis of IE. Moreover, the effects of anti-microbial therapy in cases of NGS-confirmed disease compared to standard therapy will also be compared. NGS has the potential to identify the genotypic characteristics of the pathogen, which is important to determine its virulent potential.

## 4. Serologic Tests

Serological tests should be performed when blood cultures are negative after 48 h. One of the micro-organisms that serological tests are used to diagnose is *Coxiella burnetii*. The microbiological criteria for the diagnosis of IE in the ESC criteria in 2023 are immunoglobulin G (IgG) antibody titers against Coxiella burnetii IgG ≥ 1:800 and indirect immunofluorescence assays (IFA) to detect IgM and IgG antibodies against Bartonella henselael or Bartonella quintana, with IgG titers of ≥1:800 [1]. The role of serological testing is also invaluable in other diagnoses. In a conducted study, serologic methods diagnosed BCNIE in 356 patients out of 754 with negative cultures, and 354 of these patients were diagnosed with Bartonellosis and Q fever [36]. However, serologic testing is not recommended for all bacteria for which serologic tests exist. For *Chlamydia*/*Chlamydophila* and Legionella infections, serologic testing is not recommended due to the high likelihood of a patient’s previous contact with these micro-organisms. This is associated with a high risk of false-positive results.

## 5. Histo-Pathology of Heart Valves after Cardiac Surgery

Cardiac surgery is performed in approximately 22.5–51.2% of patients [9,37]. Material collected during cardiac surgery should be subjected to histo-pathological and microbiological examination. It is even more important in cases of BCNIE, as it can deliver the causative micro-organism and improve the antibiotic therapy. However, it should be noted that cultures from cardiac valve tissues have a low sensitivity of 6–26% [38]. The histo-pathology of infected valves can give the diagnosis of IE, even without micro-ogranisms demonstrated in tissues. The presence of necrosis and inflammation, which is an indicator of the severity of inflammation in IE patients, is detected using conventional hematoxylin and eosin staining. In the case of fungi and micro-organisms for which culture is more challenging, there are specialized stains: Warthin–Starry silver for *Bartonella* spp., periodic acid Schiff for the detection of *T. whipplei* or methenamine silver in fungi are used [39]. However, the use of these can be justified when there is a suspicion of such bacteria according to the local epidemiology. Moreover, the histo-pathological analysis can be useful as it may reveal the non-infectious causes of endocarditis, including auto-immune and neoplastic causes.

Molecular testing is also possible from excised tissues or heart valves, especially considering the fact that bacterial DNA is even more abundant in tissue samples compared to blood or plasma.

In the study which retrospectively assessed the histo-pathology criteria of IE in valve samples, it was demonstrated that histo-pathology established the diagnosis of IE in 37 out of 49 (75%) cases of blood culture-negative IE and 58 out of 69 (84%) cases of blood culture-positive cases [40].

## 6. Molecular Techniques

**Figure 3:**
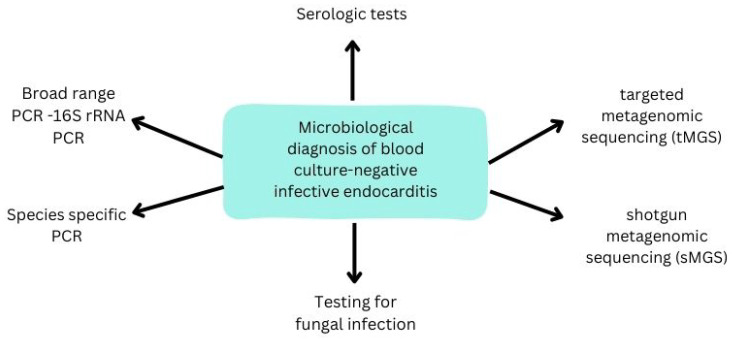
Microbiological diagnosis of negative blood-culture infective endocarditis.

Specific molecular methods that can help diagnose IE include organism-specific PCR assays that detect a specific micro-organism, broad-range PCR with amplification primers targeting the bacterial 16S rRNA gene, targeted metagenomic sequencing (tMGS), and shotgun metagenomic sequencing (sMGS), in which all genomic DNA sequences are extracted from a blood or emission sample. The sensitivity and specificity of these techniques are higher for explanted tissue than for blood or plasma [41]. They may provide a micro-organism diagnosis in BCNIE (Figure 3). Molecular analysis seems to be the only method with consistent results in determining the etiology of BCNIE. What is also important is the fact that molecular analyses are much faster than culture diagnoses, especially in the case of fastidious micro-organisms.

Methods for the amplification and sequencing of ribosomal RNA genes (16S rRNA for bacteria and 18S rRNA for fungi) began to be used in the diagnosis of BCNIE. The materials used for these methods in IE are plasma or blood samples and heart valve tissue. However, due to the small biomass of pathogens and, thus, not enough bacterial DNA, blood as a diagnostic material is not as good as heart valve tissue. Valves have more bacterial genetic material compared to whole blood and plasma [42]. For comparison, the sensitivity of the Bartonella PCR test in blood and serum was 36%; in heart valve tissue, it was 92% [43]. The PCR method is mainly used for IE in patients after surgical treatment. Compared to tissue culture, the 16S rRNA PCR testing of cardiac tissue in IE patients treated surgically is a more effective method [44]. The specific PCR only amplifies the DNA material of specific bacteria. Hence, one test is designed for only one micro-organism. Broad-range PCR is used to detect and differentiate bacteria. After detecting the bacterial DNA, the hypervariable regions of the 16S and 18S rRNA genes of bacteria are amplified. The 16S DNA gene contains six conserved sequences common to all bacteria. However, variable regions are specific to certain bacterial species. Thus, it is possible to differentiate species by using this method. However, even the smallest contamination or the wild background bacterial DNA (which will attach itself to DNA polymerase) can lead to false positive results by inappropriately amplified and identified background sequences [45]. Another disadvantage of these approaches is that the DNA from the causative agent must be present at a relatively high concentration to be properly detected [45].

To date, many studies have been conducted that confirmed the superiority of PCR testing against blood and valve cultures in IE. Boussier et al. showed that two-step broad-range PCR improves the diagnostic yield for BCNIE by up to 37.5% when compared to heart valve culture [46]. In a recent study, 41% of patients with BCNIE were found to be positive by using 16S rDNA PCR [47]. 16S rDNA PCR and sequencing assays can also be useful diagnostic tools for patients who have a positive blood culture test due to suspected skin commensal contamination and have undergone prior antibiotic therapy [48]. In those cases, the 16S rDNA PCR method can reduce the false-positive results and the risk of unnecessary IE treatment with broad-spectrum antibiotic therapy or an inappropriate antibiotic. Baddour et al. showed that 17% of patients (who had skin commensals identified in previous blood cultures) tested via the 16S rDNA PCR method were found to have species such as T.whipplei and C.burnetti that do not respond to treatment with empirical antibiotic regimens for IE treatment from the guidelines [49].

What should also be noted is that 16S rRNA PCR can detect the causative micro-organism upon post-operative examination in 83% of patients, with definitive IE successfully treated using antibiotic therapy. What is crucial is the fact that the high specificity and sensitivity of 16S rDNA PCR sequencing involves both native and prosthetic valve endocarditis [50]. Therefore, the technique cannot be used to monitor infections or determine the duration of post-operative therapy. However, the pathogen can be detected using this method even with prior use of inappropriate antibiotics. It can give a chance for administrating the antibiotic with the proper spectrum for the causative agent of IE.

To date, numerous studies have been conducted to demonstrate the utility of PCR in the diagnosis of IE. A study by Peeters et al. (involving 127 patients) showed that the sensitivity for detecting the causative micro-organism was low for valve cultures (26%) and high (87%) for blood cultures [51]. However, the sensitivity of 16S rRNA PCR for valves was higher compared to valve culture. The most favorable impact of molecular testing came from additional diagnostic value: an additional diagnostic value in 21% and a therapeutic impact in 10% of the included patients [51]. In another retrospective study, the additional diagnostic value of broad-range PCR testing was applied to resected heart tissue or swabs from resected heart tissue regarding BCNIE [52]. According to the results, the pathogen was identified in 21.5% of tissues by using broad-range PCR as an additional application [52]. In a one-center experience from Germany, broad-range 16S and 18S rDNA PCR and sequencing testing were used for the identification of heart tissues derived from valve replacements. It changed the course of antibiotic therapy in 7 out of 46 patients [53]. In a study by Rampini et al., the analysis of blood bacterial culture with the use of PCR was performed prospectively for 231 specimens, with a negative result for routine bacterial culture. The sensitivity of wide-range PCR was 81.9%, and specificity was 93.8%, where the concordance between culture and PCR for 16S rDNA was >90% [54]. Moreover, the obtained data also showed that 16S rRNA gene PCR is particularly useful for the identification of bacterial pathogens in patients pretreated with antibiotics [54]. Moreover, a study of valve samples from 36 patients undergoing cardiac surgery showed that broad-spectrum PCR detected micro-organisms in valves significantly more often (*n* = 14; 53.8%) compared to valve culture (*n* = 8; 30.7%) (*p* < 0.001) [55]. Rodriguez-Garcia et al. demonstrated that PCR testing heart valves using 16S rDNA is more useful when compared to tissue culture in patients undergoing surgery [56]. By using 16S rDNA PCR, the causative micro-organism of IE was identified in 36% of blood culture-negative cases [56]. One of the largest studies of the microbiological findings on valves was the retrospective study by Johansson et al. on 272 patients with IE. Its purpose was to investigate the diagnostic benefits of the 16S-rDNA PCR method. The concordance between blood cultures and 16S analysis was found to be 77%. In addition, the use of the method provided a diagnostic benefit in 9.0% of episodes [57]. Thus, it has been demonstrated that it may be important to perform both blood cultures and sequencing on valves taken during IE cardiac surgery. Thus, 16S analysis can help determine both the microbial etiology in BCNIE cases and to resolve discrepancies between valve and blood cultures [57].

Interestingly, molecular testing can also be useful in showing bacteria susceptibility. In the prospective study by Mularoni et al., a molecular anti-biogram of 17 valve specimens was performed, along with traditional culture-based anti-microbial susceptibility testing. The molecular anti-biogram was based on genes encoding for multi-drug resistant mechanisms. It showed 100% concordance with anti-microbial susceptibility testing [58].

Although the PCR technique in whole blood samples is not as specific as in solid tissue, it can also help to identify the causative agent of IE. It does not require separate testing with specific agents for specific groups, as is the case for various blood cultures [59]. In the study, the real-time PCR of 16S rRNA was performed on 20 whole blood samples with definitive IE. A total of 13 of these were positive using the real-time PCR technique. The isolated bacteria were *Enterococcus faecalis*, *Streptococcus gallolyticus*, *Streptococcus mutans*, *Streptococcus sanguinis*, *Streptococcus salivarius*, and *Staphylococcus aureus* [59].

From the analysis of the explanted heart valve samples from 151 patients from hospitals in the UK and Ireland, PCR was shown to detect the causative micro-organism in BCNIE [60]. The most frequently detected micro-organism was *Streptococcus* spp. Interestingly, there was a relatively high number of IE cases with *Bartonella* spp., and *Tropheryma whipplei* etiologies were identified via PCR [60]. Many case reports of rare causes of IE have also been described, in which only 16S rRNA PCR performed on explanted valve tissue yielded a diagnosis and appropriate antibiotic therapy. The case of chronic Q fever PVE is one example that is difficult to diagnose without this method because blood cultures are difficult to culture [61]. The other micro-organisms that are difficult to culture and identify by using broad-range PCR are *Corynebacterium jeikeium* and *Capnocytophaga canimorsus* meningitis [62,63].

Studies have also been conducted to explore the identification of the 16S rRNA PCR method in the diagnosis of sepsis. The 16S rRNA PCR method has also shown good diagnostic results in samples such as cerebrospinal fluid [64]. However, in the diagnosis of neonatal sepsis in newborns, the study showed a low sensitivity of 16.6% and a specificity of 97.8% compared to blood culture [65]. Similar to the patients with IE, in septic patients, other methods, such as metagenomic sequencing analysis, have been studied [66,67]. Beyond improving the diagnosis of infection, they can potentially provide novel genetic insight into microbiological fluctuations during septic progression.

Since not all IE patients undergo cardiac surgery and, as mentioned above, 16S rRNA PCR has the greatest utility in valve tissue samples, there is a need for something to diagnose BCNIE in blood samples. Next-generation sequencing (NGS) has begun to play a role in IE diagnosis. In 2019, Blauwkamp et al. described the validation and implementation of the shotgun metagenomic sequencing (sMGS) assay (the Karius test). It detects microbial cell-free DNA (mcfDNA) in plasma [68]. The test showed a 93.7% concordance with blood cultures in a cohort of 350 patients with sepsis [68]. The study by Lamoureux et al. compared the performance of sMGS (46.5% of cases) to 16S PCR (38.8%) for bacterial detection and identification [69]. It also underlines the role and better performance of sMGS in the diagnosis of patients with infectious diseases.

The first prospective study was a pilot study evaluating targeted metagenomic sequencing (tMGS) for the early detection and identification of pathogens in IE from blood. Both whole-blood and plasma samples were subjected to nucleic acid extraction and PCR (targeting the V1–V3 region of the 16S ribosomal RNA gene), followed by next-generation sequencing using the Illumina MiSeqTM platform. The positivity rate of the tMGS assay was 66% overall and 83% in BCNIE, independent of previous antibiotic treatment [70]. Moreover, another study also demonstrated the potential usefulness of meta-taxonomy to improve the microbiological diagnosis of IE and suggest co-infection in patients with IE [71].

In a recent study, the first comparison between tMGS and sMGS was investigated in samples from 34 patients. The Karius test (sMGS) was positive in 24 out of 34 cases (71%). However, it was not significantly different from the positivity rate of tMGS (*p* = 0.41). Moreover, the results of the Karius test were consistent with the results of tMGS in 75% of cases. In conclusion, by combining the methods together (blood cultures, Karius test, and tMGS), it was possible to find a potential causative agent in 97% of patients, including 5 out of 6 with BCNIE [72].

The amplification and sequencing of RNA genes—16S rRNA (for bacteria) and 18S rRNA (for fungi)—are used to identify pathogens in plasma, whole blood, and tissues. Undoubtedly, the best results were achieved in the analysis of heart tissues, characterized by high sensitivity and specificity. The combination of traditional culture methods, histo-pathological analysis, and PCR allows for a diagnosis in the vast majority of patients with IE. Molecular techniques, such as those based on traditional blood cultures, also allow for the selection of antibiotic therapy. The amplification and sequencing of RNA genes (16S rRNA for bacteria and 18S rRNA for fungi) resulted in the identification of pathogens to a much lesser extent than in the case of tissues. This problem is solved by introducing next-generation sequencing. The combination of sMGS and tMGS for traditional culture allowed for the identification of pathogens in 97% of patients. The summary of the molecular methods is included in the Table 1.

## 7. Imaging Infective Endocarditis by Using Novel Techniques

IE can include electrodes or native or prosthetic valves. The gold diagnostic standard—TTE and TEE—focuses on visualizing any complications of the valves in IE. However, it is also possible to image IE-induced inflammation using newer techniques. The pathophysiology of IE involves inflammation and bacterial proliferation, which leads to the formation of vegetation made of fibrin, micro-organisms, platelets, and inflammatory cells [1]. In patients with prosthetic heart valves and implanted pacing devices, the criteria for diagnosing IE have a lower value and a high rate of false-negative results. Therefore, since 2015, the diagnostic criteria for suspected prosthetic valve IE (PVE) in patients with CDRIE include positron emission tomography/fluoro-deoxygenase PET/CT (^18^F-FDG PET/CT), along with single-photon emission tomography/technetium oxym99m-hexamethylpropyleneamine (^99m^Tc-HMPA) (^99m^Tc-HMPAO-SPECT/CT) [1,5,73].

One of the methods used is ^99m^Tc-HMPAO-SPECT/CT, which is based on the intra-cellular labeling of isolated white blood cells with a ^99m^Tc-HMPAO complex and their imaging at a specific time after tracer administration [74,75,76]. The test is performed according to a 24 h protocol, including early (30–60 min), delayed (2–4 h), and late (20–24 h) acquisition [77]. After intravenous administration, radioactively labeled white blood cells migrate first to the respiratory system and then to the liver, spleen, and reticuloendothelial tissues [76,78]. Migration is then guided by chemotactic attraction to the bone marrow and infected sites [76,78]. White blood cells are observed in various organs and in areas of the bone marrow at specific time points after intravenous administration, but the intensity of physiological uptake does not increase over time in delayed and late ^99m^Tc-HMPAO-SPECT/CT acquisitions. In contrast, at sites of infection, the opposite is true—leukocytes accumulate over a longer period of time and lead to later acquisitions. ^99m^Tc-HMPAO-SPECT/CT is considered positive for IE when there is at least one site of increased intracardiac radiolabel uptake and/or near the CIED lead. This method has high specificity and sensitivity for suspected PVE, especially when the TTE result is equivocal [79,80,81]. The effectiveness of imaging for infection may be affected by previous antibiotic therapy, the type of pathogen causing the infection, and the vascularization of the infected tissue [78]. Although the examination is time-consuming, the technique provides high precision, especially in the context of differentiating sterile and infectious intramural morphological lesions [82].

The diagnostic accuracy of this technique was also evaluated via the intra-operative findings in patients with PVE [83]. Patients with an intense signal on WBC-SPECT had a high prevalence of abscesses (83%) [83]. In a systemic review by Holcman et al., the overall sensitivity of ^99m^Tc-HMPAO-SPECT/CT was studied to be 60–93.7%, specificity was 88–100%, the negative predictive value (NPV) was 84.6–93.9%, and the positive predictive value (PPV) was 74–100% [84]. What is crucial is the fact that the ^99m^Tc-HMPAO-SPECT/CT examination is able to reliably exclude CDRIE in patients with fever, sepsis, or a suspicion of IE [85,86]. Unfortunately, prior anti-microbial therapy appeared to be associated with a higher rate of false-negative HMPAO-SPECT/CT results (OR, 4.63; 95% CI, 1.41 to 15.23, *p* = 0.01) [87]. In this prospective study, the technique showed an accuracy of 81.95%, a specificity of 86.92%, a sensitivity of 73.33%, an NPV of 84.96%, and a PPV of 76.39% [87].

^18^F-FDG PET/CT is based on the accumulation of a radioactively labeled glucose analog (^18^F-FDG) in cells with a large number of metabolically active glucose transporters expressed on their cell surface—activated inflammatory cells (macrophages, leukocytes, and lymphocytes) [88]. Heterogeneous uptake within the heart may be related to infection [89]. In addition, ^18^F-FDG PET/CT allows for a quantitative evaluation of extracardiac septic foci, which are complications of IE and represent small criteria for IE diagnosis. However, the diagnostic accuracy of this technique depends on several factors, mainly on proper preparation. In order to suppress the natural uptake of radiolabel in the myocardium, a low-carbohydrate and high-fat diet is used for 12–24 h prior to the examination, followed by a period of fasting [89].

In addition, 15 min before the administration of the radiolabel, intravenous heparin is administered. The acquisition itself during the study is short and should be performed 60 min after the administration of the radiolabel. The standardization of imaging protocols and the procedure are key to obtaining reliable data. It is also worth remembering that numerous other lesions can mimic IE-like uptake. These include primary and metastatic cardiac tumors, vasculitis, reactions after cardiac surgery, and reactions to foreign bodies [90].

Another imaging method based on nuclear diagnostic methods is [^18^F]-FDG PET/CT. It is based on the accumulation of a radioactively labeled glucose analog (^18^F-FDG) in cells with a large number of metabolically active glucose transporters expressed on their cell surface—activated inflammatory cells (macrophages, leukocytes, and lymphocytes) [88]. Heterogeneous uptake within the heart may indicate infection [89]. In addition, [^18^F]-FDG PET/CT allows for the assessment of extracardiac septic foci, which are complications of IE and small criteria for IE diagnosis. However, the diagnostic accuracy of this technique depends on several factors, mainly on the proper preparation for the technique. A low-carbohydrate and high-fat diet for 12–24 h before the test, followed by a period of fasting, is used to inhibit the natural uptake of the radiolabel in the myocardium [89]. In addition, intravenous heparin should be administered 15 min before the administration of the radiolabel. The acquisition itself during the study is short and should be performed 60 min after the administration of the radiolabel. Unfortunately, the test can produce false-positive images because many other lesions can mimic IE-like uptake. These include primary and metastatic cardiac tumors, vasculitis, reactions after cardiac surgery, and reactions to foreign bodies [90]. The use of [^18^F]-FDG PET/CT for the diagnosis of IE in patients with suspected PVE (Class I) and CDRIE when TEE does not provide a clear answer (Class IIB) [1] should be considered. Performing the test leads to a more accurate reclassification of patients with suspected PVE [91,92]. In contrast, the use of the test is limited in the diagnosis of NVE [1]. Obstacles to the detection of small vegetations include continuous cardiac motion during the study and the low spatial resolution of PET/CT imaging, reaching 5 mm [93]. A meta-analysis of 15 studies with 333 cases evaluating the PET method in PVE confirmed an overall sensitivity of 86% and a specificity of 84% [94]. In contrast, for CDRIE, efficacy was estimated to be 86.67–93%; accuracy was 62.5–100% for specificity, 30.8–100% for sensitivity, 66–100% for PPV, and 75–100% for NPV [84]. Unfortunately, prior antibiotic therapy can reduce the effectiveness of the test and produce false-negative imaging results [95,96,97].

An undoubted limitation of echo-cardiography is that a negative result does not exclude IE, especially in people with a moderate or high risk of endocarditis. Vegetations may not be visible if they are very small or if imaging is difficult, such as in patients with more than one artificial heart valve. The lack of a conclusive result usually requires repeating the transesophageal echo-cardiographic examination. Moreover, it may be impossible to identify small abscesses, especially during the very early stages of their development in the area of the mitral valve with severe mitral annular calcification. Additionally, echo-cardiography may provide false positive results, where additional structures, such as non-infectious thrombi, fibrin strands, pannus, and prominent endothelialized sutures, may resemble vegetations.

Current nuclear medicine techniques, primarily PET/CT with FDG, added to standard visualization techniques, increase diagnostic sensitivity from 52–70% to 91–97% without compromising specificity and allow for the identification of primary infection source and/or septic embolism [98]. However, a significant limitation of the method is the differentiation between device infection and nonspecific inflammation. In order to improve the differentiation between infection and inflammation, new tracers are currently being tested in research. In the case of [18-F]-FDG PET/CT, which is currently the most commonly performed nuclear imaging modality in IE, prolonged fasting and dietary preparation can be considered a limitation. This is especially true for patients with diabetes for whom there are no dedicated dietary guidelines. In the future, a new tracer may enter clinical practice, which will provide high specificity and sensitivity. Moreover, it should allow for the differentiation between inflammation and infection.

## 8. Future Diagnostic/Research

**Figure 4:**
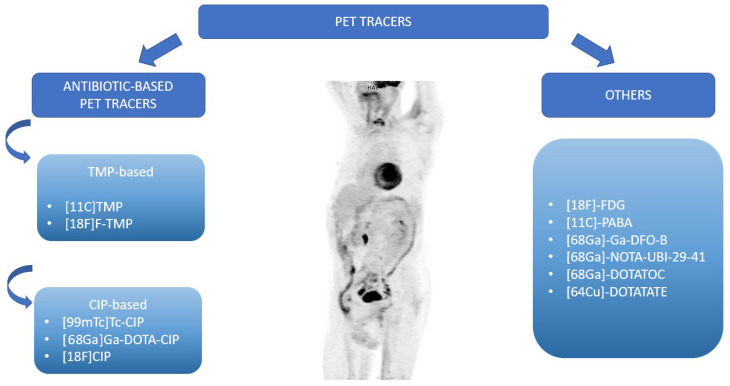
PET tracers have potential to be used in the clinical practice and diagnosis of IE.

Future research on infective endocarditis should focus on a better diagnosis of BCNIE. The identification of the pathogen responsible for IE leads to the implementation of effective treatment. This can be achieved through the standard use of 16S/18S PCR diagnostics and increasing its availability, as well as the further development of nuclear medicine imaging in IE. The ideal tracer would not require a special diet or the administration of additional drugs (such as [^18^F]-FDG), and the acquisition time for the test would be short (unlike ^99m^Tc-HMPAO-SPECT/CT). In addition, it should be stable and simple to manufacture so that storage conditions would not affect its effectiveness. The ideal radiolabel would be specific only to the infection of a particular tissue, in this case, the endocardium. Several studies have appeared that point to certain tracers that are being considered for infection detection in molecular imaging. The potential tracers are demonstrated in Figure 4.

To date, several early-phase studies with antibiotic-based PET radioactivity have been published. Most of these are currently in preclinical development; they can be divided into two subgroups: structurally modified and unmodified antibiotic radioactivity [99]. Most of them are also not specific to endocardial infection, only indicating inflammation nonspecifically. However, as with [^18^F]-FDG PET/CT, this property can also be used to identify the source of infection, as well as IE complications.

One of the potential radiolabels is based on trimethoprim (TMP). Trimethoprim-based radiolabels can be diagnostically helpful in patients with chronic infections (such as bone and bone marrow infections) caused by bacteria that are both sensitive and resistant to TMP. The biodistribution of [^11^C]TMP differs from the commonly used metabolic [^18^F]-FDG tracer. The [^11^C]TMP tracer is described in an article by Mark A. Sellmyer et al. [100]. The authors emphasize that this tracer may be applicable in cases where bacterial infection cannot be distinguished from benign inflammation or tumor on the basis of anatomical imaging or other standard nuclear imaging techniques. The sensitivity of [^11^C]TMP in detecting acute bacterial infection seems to be promising due to the low uptake of the background radiolabel in many tissues (blood, heart, muscle, lung, spleen, skin, brain, bone, stomach, and pancreas).

Lee et al. described the mechanisms of pathogen resistance to TMP and found that antibiotic-insensitive bacterial species capture [^11^C]TMP in a similar manner to sensitive species. This underscores the potential of radioactively labeled TMP to image a variety of pathogens, regardless of their resistance status to TMP [101]. A phase 1 clinical trial (NCT03424525) using [^11^C]-Trimethoprim PET/CT in patients with suspected bacterial infections is currently underway. Up to 30 participants will be recruited and will participate in two different imaging cohorts. The biodistribution cohort will include up to five patients. The dynamic cohort will include up to 25 patients, who will undergo a 60 min (approximately) dynamic scan followed by static scans after [^11^C]-trimethoprim injection. Patients who are selected for antibiotic treatment based on clinical indications may also undergo a second PET/CT scan with [^11^C]-trimethoprim to collect pilot data on changes in biodistribution and tracer uptake during infection therapy.

Sellmyer et al. also described the use of another radiotracer based on thrimethoprim—[^18^F]fluoropropyl-trimethoprim. In a murine model, they identified bacterial infection by *E. coli* and *S. aureus* with this tracer, while no increased signal was obtained in the same animal for chemical inflammation and for breast cancer [102]. The tracer uptake in infected tissues by the bacteria was shown to be approximately double compared to the controls. Currently, there is a registered clinical trial (NCT04263792) recruiting for phase 1, the aim of which is to evaluate the [^18^F]fluoropropyl-trimethoprim ([^18^F]F-TMP) PET/CT biodistribution and kinetics of a radiotracer to see how it is taken up in sites of active infection.

Koźmiński et al. described the experimental results of another potential radiolabel based on synthesized ciprofloxacin [103]. The radioconjugates contained technetium-99m ([^99m^Tc]Tc-CIP) or gallium-68 ([^68^Ga]Ga-DOTA-CIP). Their physicochemical properties (stability and lipophilicity) and biological properties (testing for binding to *Staphylococcus aureus* and *Pseudomonas aeruginosa*) were investigated. The authors observed that [^99m^Tc]Tc-CIP was a better vector for Gram-positive bacteria, as the uptake of this radioconjugate by *S. aureus* was significantly higher than by *P. aeruginosa*. They also noted that the more lipophilic CIP-based radioconjugates were more efficiently bound by bacterial cells [103]. However, this study referred to diabetic food infections, and currently, no data have been published on infective endocarditis with these tracers. Another ciprofloxacin-based PET tracer, [^18^F]ciprofloxacin, was shown not to be suitable as a bacteria-specific infection imaging agent for PET [104]; as this was demonstrated in a clinical trial, no other studies further investigated this tracer. There are two ^68^Ga-labeled infections targeting ciprofloxacin radiotracers that were studied by Satpati et al. [105]. Their efficacy has been investigated in vitro, showing that they could potentially discriminate between bacterial infection and inflammation in vivo [105]. Thus, they are worthy of further detailed investigation as infection imaging agents at the clinical level.

Another tracer that could potentially be used to detect infections, including IE, is 6-[^18^F]fluoromaltose, described by Gowrishankar et al. The researchers created this tracer and demonstrated that it could potentially be used in vivo to image bacterial infections. This PET tracer is captured specifically by bacteria and minimally by human cells. Importantly, it appears to have the potential to distinguish between infection and inflammation (unlike other tracers). Unfortunately, there are currently no other studies on this interesting topic [106].

Imaging using the differences between human and bacterial cells may also be an interesting direction for future research. Bacteria synthesize folic acid, and the tracer was produced by incorporating para-aminobenzoic acid (PABA) into tetrahydrofolate via a bacterial synthetase. Ordonez et al. showed that [^11^C]PABA was able to detect the sites of infection caused by *E. coli* and *S. aureus* in a rabbit model of myositis with a signal four–five times higher compared to sterile inflammation in contralateral muscle [107]. [^11^C]PABA was also able to detect the site of infection in a prosthetic joint infection model caused by MRSA [106]. In addition, a preliminary study showed that the use of [^11^C]PABA is safe, well tolerated, and rapidly eliminated by the kidneys, indicating its potential clinical use for localizing sites of infection in humans.

Another difference between human and bacterial cells is siderophores, which are iron-specific chelators recognized by specific transporters. The replacement of iron with gallium-68 without loss of bioactivity is possible [108]. By using various microbial cultures, [^68^Ga]Ga-DFO-B uptake has been tested in vitro. PET/CT images of animal models of infection were performed and showed the high and specific accumulation of [^68^Ga]Ga-DFO-B in both *P. aeruginosa* and *S. aureus* infections with excellent image contrast. In contrast, in sterile inflammation, heat-inactivated *P. aeruginosa* or *S. aureus*, and *Escherichia coli* lacking DFO-B transporters, no uptake was observed [108].

Another potential radioactive tracer has been described by Vilche et al. The researchers used ^68^Ga-NOTA-UBI-29-41 to analyze its in vivo biology during infection and inflammation and in healthy mouse models to assess its potential as a tracer in PET imaging. They showed that the radiolabel distinguishes bacterial infection from sterile inflammation. Moreover, the foci of *S. aureus* infection were clearly defined as early as 30 min after tracer administration [109]. Mukherjee et al. also demonstrated the feasibility of developing a kit to prepare this radio-pharmaceutical quickly and easily for clinical use [110]. ^68^Ga-NOTA-UBI-29-41 was also injected intravenously into three patients with suspected infection. The uptake of the radio-pharmaceutical clearly demarcated the foci of infection from normal nontarget tissues, making it another potential radio-pharmaceutical for use in imaging infection.

Several registered clinical trials testing further potential radio-pharmaceuticals are currently underway. The DOTENDO trial (NCT05183555) aims to evaluate whether somatostatin receptor PET imaging is feasible for the diagnosis of infective endocarditis. ^68^Ga-DOTATOC (^68^Ga-edotreotide) has previously been used in the diagnosis and follow-up of neuroendocrine tumors. ^68^Ga-DOTATOC binds with high affinity to the somatostatin receptor. The other two studies, NCT05432427 with ^64^Cu-DOTATATE and NCT05446376 with 68 gallium citrate, also aim to evaluate the diagnostic efficacy of these tracers in the diagnosis of infective endocarditis.

## 9. Conclusions

Recent years have seen developments in the diagnosis of bacterial infections, including IE, using new molecular methods. Early studies show that they can effectively help establish a diagnosis. However, they are currently not widely used due to their cost. It is likely that further research will lead to the more widespread use of these methods, leading to lower costs. It is important to unambiguously establish the diagnosis of IE and identify the responsible micro-organism to use the appropriate and targeted antibiotic therapy or not if this is unnecessary. A perfect diagnostic method for IE should ensure quick (preferably within a couple of hours) and very precise results regarding the identification of the pathogen and its susceptibility to antibiotics independent of previous anti-microbial treatment. The method should be cost-effective, allow the recognition of all possible micro-organisms that can cause infective endocarditis, be easy to implement, and should not require very sophisticated measures or highly trained personnel. All of the above would allow a global unification of diagnostic methods. The existing standards are based mainly on blood cultures because of this wide availability and low costs. Another advantage is that they allow for the identification of a wide variety of bacteria causing IE. The disadvantages include the various times required for the blood to be drawn to obtain the results and low sensitivity. New methods provide much quicker results; however, sometimes, they allow for the identification of only a limited number of species of micro-organisms. Further disadvantages include the necessity of specialized equipment, highly trained personnel, and costly measures to perform the tests. However, the use of some methods is supported by very limited data; as a result, they still cannot be widely used.

## Figures and Tables

**Table 1:** The characteristics and benefits of molecular methods used in the diagnosis of IE [72].

Molecular Method	Short Characteristic/Benefit
Organism-Specific PCR Assays	Detects specific microorganisms for which test is dedicated
Broad-Range PCR with 16S rRNA Gene	Detects bacterial DNA in blood or plasma, has higher sensitivity and specificity for explanted tissue than for blood or plasma
Targeted Metagenomic Sequencing (tMGS)	It is less cost-consuming comparing to the sMGS and the overall positivity was not from sMGS [72].
Shotgun Metagenomic Sequencing (sMGS)	Detects microbial cell-free DNA, it can also detect fungal species and resistent microorganisms, contrarly to tMGS [72].

## Data Availability

No new data were created or analyzed in this study. Data sharing is not applicable to this article.
